# Evaluation of the chicken transcriptome by SAGE of B cells and the DT40 cell line

**DOI:** 10.1186/1471-2164-5-98

**Published:** 2004-12-21

**Authors:** Matthias B Wahl, Randolph B Caldwell, Andrzej M Kierzek, Hiroshi Arakawa, Eduardo Eyras, Nina Hubner, Christian Jung, Manuel Soeldenwagner, Manuela Cervelli, Yan-Dong Wang, Volkmar Liebscher, Jean-Marie Buerstedde

**Affiliations:** 1 Institute of Molecular Radiobiology, GSF, Ingolstädter Landstr. 1, D-85764 Neuherberg, Germany; 2 Laboratory of Systems Biology Institute of Biochemistry and Biophysics, Polish Academy of Sciences, Pawinskiego 5a, 02-106 Warszawa, Poland; 3 Research Group of Biomedical Informatics, IMIM/Universidad Pompeu Fabra/Centre de Regulacio Genomica, E08003 Barcelona, Spain; 4 Institute of Biomathematics, GSF, Ingolstädter Landstr. 1, D-85764 Neuherberg, Germany; 5 Stowers Institute for Medical Research, 1000 E. 50th Street, Kansas City, MO 64110, USA

## Abstract

**Background:**

The understanding of whole genome sequences in higher eukaryotes depends to a large degree on the reliable definition of transcription units including exon/intron structures, translated open reading frames (ORFs) and flanking untranslated regions. The best currently available chicken transcript catalog is the Ensembl build based on the mappings of a relatively small number of full length cDNAs and ESTs to the genome as well as genome sequence derived in silico gene predictions.

**Results:**

We use Long Serial Analysis of Gene Expression (LongSAGE) in bursal lymphocytes and the DT40 cell line to verify the quality and completeness of the annotated transcripts. 53.6% of the more than 38,000 unique SAGE tags (unitags) match to full length bursal cDNAs, the Ensembl transcript build or the genome sequence. The majority of all matching unitags show single matches to the genome, but no matches to the genome derived Ensembl transcript build. Nevertheless, most of these tags map close to the 3' boundaries of annotated Ensembl transcripts.

**Conclusions:**

These results suggests that rather few genes are missing in the current Ensembl chicken transcript build, but that the 3' ends of many transcripts may not have been accurately predicted. The tags with no match in the transcript sequences can now be used to improve gene predictions, pinpoint the genomic location of entirely missed transcripts and optimize the accuracy of gene finder software.

## Background

The definition of transcription units within a finished genome sequence in higher eukaryotes is challenging and relies on genome mapping of cDNAs and ESTs backed up by theoretical gene finder algorithms. An increasing number of gene sequences from model organisms have made the prediction of well conserved ORFs easier, but less conserved coding and untranslated regions are difficult to detect. The best way to unambiguously define transcription units is full length cDNAs, but large scale projects are expensive in terms of labor and costs. One also needs to bear in mind that some cDNAs elude detection, because unusual secondary structure or toxicity inhibits reverse transcription or cloning.

SAGE investigates the transcription profile of a given cell sample by large scale sequencing of short cDNA tags derived from the bulk mRNA [1,2]. Whereas tag mapping to the cDNA and genome databases of the organism indicates the type of expressed genes, the prevalence of individual tags within the library reflects their relative levels of expression. Since SAGE tags are only short sequences, they can be collected more easily in higher numbers than ESTs and full length cDNA sequences. The potential of SAGE to discover new or better define already known transcription units is particularly advantageous in situations where the entire genome sequence of an organism has been determined, but gene predictions based on theoretical algorithms and the mapping of a relatively small number of EST and cDNA sequences remain tentative. LongSAGE generates longer tags of 21 bases as compared to the classical SAGE protocol and is therefore better suited for the unambiguous assignments of tag to genome sequences [3,4].

Cellular and molecular features of early B cell development [5] and lymphoma formation [6,7] have been extensively studied in the chicken. Gene expression signatures of primary bursal B cells, pre-neoplastic and neoplastic lymphoma cells were collected by microarray hybridizations in a first attempt to identify genes up- or down-regulated during myc-induced B cell lymphoma development [8].

The whole chicken genome including a genome scale transcript build from Ensembl [9] and a collection of bursal full-length cDNAs [10] have recently been released. We describe here the mapping of large collections of SAGE tags from bursal lymphocytes and DT40 to these reference datasets to evaluate the quality of the transcript build. Furthermore, the transcription profiles of bursal cells and DT40 as defined by this first SAGE analysis in the chicken should lead to a better understanding of B cell transformation and facilitate the selection of candidate genes for disruption in DT40 [11].

## Results and Discussion

### Generation of SAGE tag libraries and SAGE tags collections

Two SAGE libraries, named busage and dt40sage, were made from the bursa of Fabricius and DT40 cells using the LongSAGE technique which generates tags of 21 nucleotides in length and therefore decreases the likelihood of ambiguous matches [3,4]. Of the 129,568 tags collected, about equal numbers were derived from the busage and the dt40sage libraries respectively (Table 1). In total 38,212 unitags were derived from the SAGE tags of both libraries. The library from bursal cells and the DT40 cell line seem to be similar with regard to the number of extracted unitags and the average counts of matching SAGE tags. Underlying a standard binomial model, one would expect to find a special Unitag among the busage tags or dt40 tags with a probability of 95% at least once if the relative abundance of this unitag among all busage tags or dt40 tags is at least 4.55 * 10^-5 ^or 4.69 * 10^-5 ^respectively.

**Table 1:** SAGE and unitag collections

	***SAGE tags***	***Unitags***	***Average frequency of matching SAGE tags within library***	***Average count of SAGE tag per unitag***
Busage	65,798	24,064	2.73	4.63
Dt40sage	63,770	21,308	2.99	4.97
Total	129,568	38,212		3.39

#### Tag to gene assignment using bursal cDNAs, the Ensembl transcript build and the genome sequence

Successful mapping of SAGE tags to reference sequences is influenced by the quality of the sequences, the complexity of the reference sequence datasets and the prevalence of polymorphisms within the tag sequences. It was therefore decided to first search for matches within a bursal cDNA collection which represents the best possible reference dataset, as it was derived from the same tissue and genetic background as the busage library. Subsequently, unitags were mapped to the Ensembl transcript build and finally the chicken genome sequence. Unitags found in a previous dataset were not searched for any more in the next. To facilitate the searches, candidate tags starting with the CATG tetra-nucleotide were extracted from each reference dataset prior to analysis.

As expected the highest rate of matching to total candidate tags was found for the bursal cDNA collection (3,030 of 26,044 candidate tags matched unitags), followed by the Ensembl transcript build (2,934 of 208,048) and the genome (14,505 of 9,091,924) (Table 2). Some unitags mapped more than once within a dataset making an unambiguous assignment difficult. In comparison to the complexity of the dataset, multiple hits occurred more frequently in the bursal cDNA (33/26,044; 0.0012%) and Ensembl dataset (637/208,048, 0.0031%) than in the genome (1,003/9,091,924; 0.0001%). This can be explained if these transcript collections are not completely normalized or if there is bias for certain sequence motifs within gene transcripts. Manual analysis of the bursal cDNAs revealed that most of the multiple unitag matches were due to alternative processing of transcripts originating from the same locus (data not shown). A relatively large fraction of unitags (17,743/38,212; 46.4%) did not match to any reference dataset. It is currently impossible to analyze this in more detail, but sequencing errors, polymorphisms and positions of tags on exon/exon boundaries are likely to explain the missed hits [12]. Non-matching unitags have a significantly lower average count of SAGE tags than matching unitags (2.1 versus 4.65) suggesting that they either over-represent lowly expressed genes or are artifacts of the SAGE technique.

**Table 2:** Unitag mapping to reference datasets

	***Dataset matches of unitag***	***Unitags***	***Candidate tags***	***Average count of SAGE tags per unitag***
Bursal cDNA		3,030	26,044	6.89
	1	2,997		6.90
	> 1	33		5.15

Ensembl transcript build		2,934	208,048	9.93
	1	2,275		10.60
	> 1	659		7.63

Genome		14,505	9,091,924	2.84
	1	13,427		2.83
	> 1	1,078		2.91

Total matching		20,469		4.45
Non-matching		17,743		2.17

SAGE tags are expected at the position of the *NlaIII *site closest to the polyA tail of the transcript, but alternative transcript processing as well as incomplete *NlaIII *digestion or internal priming can produce upstream tags. Indeed, when the positions of the matching candidate tags were analyzed for bursal cDNA transcripts, about 40% of the tags matched to non-last positions (data not shown).

#### Mapping of tags to the genome

Most interesting from the perspective of gene discovery are the 13,427 unitags without transcript match, but with a single match in the genome (Table 3). When the positions of these tags within the genome were correlated with the positions of the Ensembl transcripts, 1,637 fell within annotated transcript boundaries indicating that they are located on missed or incomplete exons. To see whether the remaining tags were located in the neighborhood of already identified transcripts, the numbers of tags falling within regions of defined length upstream and downstream of the Ensembl transcripts were determined. Indeed many tags map very close to annotated transcripts with a strong preference for the region downstream of the transcript, as would be expected, if the tag matches the missed 3' end of an annotated Ensembl gene. Since not all tags are derived from the most 3' transcript position, the tags matching immediately upstream of transcripts might indicate missed 5' exons. Some of the tags mapped close to upstream and downstream transcripts (12 at the 500 base distance limit), perhaps indicating that these transcripts belong together. At a distance limit of 5000 bases, 7,169 tags mapped into the neighborhood of annotated transcripts; 5,627 downstream, 669 upstream and 1,061 both upstream and downstream. When the distance limit was extended to 10,000 bases, the number of downstream matching tags was only marginally increased to 5,627 whereas the number of dual positioned tags more than doubled to 2,101. This indicates that at distances over 5000 bases the tag assignment to the neighboring transcripts is becoming increasingly ambiguous, and the tags might in fact correspond to entirely missed genes.

**Table 3:** Locations of unitags having a single match in genome but no transcript match

	***Unitags***	***Bases searched next to annotated Ensembl transcripts***	***Matching unitags***	***Matches only downstream of Ensembl transcripts***	***Matches only upstream of Ensembl transcripts***	***Matches upstream and downstream of Ensembl transcripts***
Total	13,427					
					
Within Ensembl transcript boundaries	1,637					
					
Outside Ensembl transcript boundaries	11,177	100	409	362	46	1
		200	732	668	64	2
		500	1,651	1,496	143	12
		1,000	2,896	2,553	262	81
		5,000	7,169	5,439	669	1,061
		10,000	8,639	5,627	911	2,101

#### Relationship of genome mapping unitags to Ensembl transcripts

To further investigate those unitags mapping close to the 5' boundary of Ensembl transcripts or within transcript boundaries to the genome, the bursal EST database [11] was searched for ESTs matching the tags in the sense strand orientation. These ESTs were then aligned to the chicken genome sequence and the neighboring Ensembl gene predictions. As many ESTs linked the SAGE tags to the Ensembl transcripts, this provided independent experimental evidence that these tags are indeed derived from non-annotated parts of these transcripts (Table 4).

**Table 4:** Analysis of unitags mapping 5' of or within Ensembl transcript boundaries. #

***Unitag***	***Ensembl ID***	***BLAST result ##***	***Supporting bursal EST***	***Unitag relationship to Ensembl transcript ###***
**Unitags mapping 5'**				

CATGCTGCTCGCACGAGCCCT	ENSGALT00000002525.1	Q9W7P7	riken1_17l12r1	Upstream 5' exon
CATGGCGGGGTTCCCGGGGCA	ENSGALT00000005092.1	PEF protein with a long N-terminal hydrophobic domain	riken1_18i20r1	Upstream 5' exon (EST supports two additional 5' exons)
CATGCTCCTGCTGCTGGCTGG	ENSGALT00000009521.1	LAC_CHICK	dkfz426_24a5r1	Upstream 5' exon
CATGAGGCACCTCCTGTTGGC	ENSGALT00000001476.1	GR78_CHICK	riken1_25c14r1	5' upstream/Exon1 (EST supports one additional 5' exon)
CATGGCCGCCCAAGGAGAGCC	ENSGALT00000004055.1	RAN_CHICK	riken1_25b20r1	5' upstream/Exon1 (EST supports one additional 5' exon)

**Unitags mapping within transcript boundaries**				

CATGTACTGGTTGTCTGTTTT	ENSGALT00000025884	HG14_CHICK	dkfz426_13h16r1	Intron 4–5
CATGCATAGAGGCTTTATTGC	ENSGALT00000021336	Aldo-keto reductase family 1 member	dkfz426_3h12r1	Intron 8–9
CATGTTGGGACTCACCACTCT	ENSGALT00000000504	No description	dkfz426_13d22r1	Intron 5–6/Exon6
CATGGTCACCCTAGTAAATAG	ENSGALT00000009677	Protein kinase C, beta type	dkfz426_38f16r1	Intron 14–15
CATGTAAAGTGTTAGCTGTAC	ENSGALT00000006857	ITF2_CHICK	dkfz426_14i24r1	Intron 8–9
CATGTTACCTGCAACCTGCTG	ENSGALT00000021577	Centromeric protein E	dkfz426_17a21r1	Intron 28–29
CATGGGATATACTGAAAATCT	ENSGALT00000009956	T-cell activation leucine repeat-rich protein	dkfz426_41d20r1	Intron 1–2
CATGGGCTGGTTGGTTTTTGT	ENSGALT00000028428	No description	dkfz426_43g3r1	Intron 2–3
CATGGTCAAGTACAACTCTTA	ENSGALT00000022583	Bcl-2-associated transcription factor	dkfz426_12n7r1	Intron 8–9

# Only a few representative examples are shown

## BLAST results are abbreviated

### Unitag aligns within an intron or exon or lies across an intron/exon or upstream sequence/exon boundary

**Figure 2:**
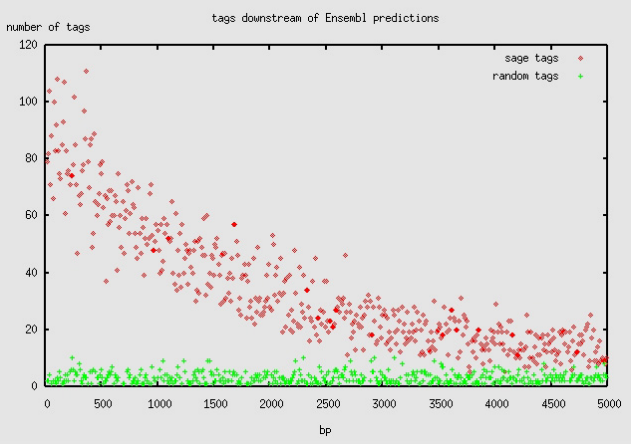
Mappings of SAGE unitags downstream of Ensembl transcripts compared to simulated genomic tags. The number of tags falling within windows of 10 bp is plotted on the y-axis whereas the distance from the 3' end of the nearest predicted Ensembl transcript is plotted on the x-axis. Sage unitags coordinates are indicated by crosses and randomly selected tag coordinates by diamonds.

To confirm that the distribution of the tags downstream of Ensembl transcripts is statistically significant, their positions were compared to the positions of simulated tags generated by randomly selecting 21 bp sequences in the genome beginning with the 'CATG' tetra-nucleotide. This comparison shows that the real tags map closer to the 3' end of the Ensembl predicted coding sequences (CDS) than the simulated tags providing strong evidence that most of the closely positioned tags are indeed related to the predicted transcripts (Figure 2).

If one summarizes the unitag to transcript mappings, 5,964 unitags map directly to transcripts, 1,637 map to not annotated sequences within the limit of the Ensembl transcripts and 7,169 map to within 5,000 bases of annotated transcript boundaries (Table 5). This leaves about 20% (4,621 out of 19,391 total) *bona fide *unitags unaccounted for which might be taken as an estimate for the percentage of genes present in the released genome sequence, but absent from the Ensembl transcript collection. Nevertheless, one needs to bear in mind that this calculation includes a number of uncertainties. It is for example possible that the 5000 base limit is too large, since only 5% of 3' UTR sequences in the human transcriptome are reported to be over 2,000 bps according to NCBI's AceView database  or that both SAGE and the gene predictions have missed a substantial number of lowly expressed transcripts. In these cases, the estimate of the percentages of missed genes would increase.

**Table 5:** Unitag mapping to transcripts

	***Unitag***	***Match to annotated transcript***	***Match to genome within boundaries of annotated transcript***	***Match next to annotated transcript using 5000 base cut-off***	***Match distant from annotated transcript***
Total	38,212				
				
Without match	17,743				
					
				
With only multiple genome matches	1,078				
					
				
With match to annotated transcripts or single genome match	19,391	5,964	1,637	7,169	4,621

#### Significant gene expression differences between bursal cells and the DT40

One of the goals of this SAGE analysis was the identification of differentially expressed transcripts between the two libraries and the significance of count differences for the busage and the dt40sage tags were calculated for each unitag. In total 629 unitags showed p values below 0.01 suggesting that the corresponding transcripts are differentially expressed in bursal cells and DT40. In contrast to this, the false discovery rate (FDR) controlling procedure of Benjamin & Hochberg would admit the first 229 genes at an FDR of 5% [13]. Twenty-five of the most significant unitags mapping to bursal cDNAs are listed in Table 6.

**Table 6:** List of genes differentially expressed in bursal cells and DT40

**Unitag**	**Busage**	**DT40sage**	**Significance**	**Sequence ID^#^**	**Best BLAST result^##^**
CATGGCAGGGGGCGGAAACCT	4	45	2.83E-10	riken1_2o24	(AAH61765) Hypothetical protein
CATGGTGAGCCAAGGTGTTGT	24	82	2.06E-9	riken1_4m1	(AAH69219) Cold inducible RNA-binding protein
CATGCAGAAATAAGCTTCTCC	45	109	4.09E-8	riken1_7b15	(Q7ZUR6) Similar to muscle-specific beta 1 integrin binding protein
CATGAGCGGGGGCAGCACTTG	118	203	5.75E-7	riken1_25p23	(Q90YW7) Ribosomal protein L4
CATGCTGGAAGAAAGAATAAC	46	114	1.92E-8	riken1_32c11	(Q9YGQ1) Peptide elongation factor 1-beta
CATGCGCTCTCCTTTTAAAAG	9	41	2.67E-6	riken1_15l3	(CAA31409) Chinese hamster asparagine synthetase
CATGGATGGCCAGCAAGTGTT	29	4	1.17E-5	riken1_4k19	(P13796) L-plastin (Lymphocyte cytosolic protein 1)
CATGTCCGTGGCATCCTTTGA	0	16	1.18E-5	riken1_24e23	(Q8BGQ8) Heterogeneous nuclear ribonucleoprotein K
CATGGCTTTGGAATATTTGAC	25	3	2.90E-5	riken1_2f9	(AAH46152) Selenoprotein P precursor
CATGGAGTCCATAACACGGCG	21	2	6.88E-5	riken1_34m12	(Q96CJ1) Testosterone regulated apoptosis inducer and tumor suppressor
CATGCAAAGTGCCCTTGGCTT	17	1	1.46E-4	riken1_10g19	(P30281) G1/S-specific cyclin D3
CATGTAAGCCAATTCTGAACC	19	1	4.09E-5	riken1_33a18	(Q8JHJ4) TNF family B cell activation factor
CATGTTGTACACACGGGCACT	11	0	5.79E-4	riken1_5g12	(Q90YB0) FEN-1 nuclease
CATGTGCCCGTGACCCCCATC	2	16	6.12E-4	riken1_4n15	(Q13200) 26S proteasome non-ATPase regulatory subunit 2
CATGTCGTGCTCTGTGCCTCC	5	26	9.28E-5	riken1_2i9	(Q90W60) XNop56 protein
CATGCTTTCTGCTTTGACTTT	21	4	9.42E-4	riken1_12p16	(P22794) Ecotropic viral integration site 2A protein
CATGTTTGTGCATAGCTGTCC	5	28	1.17E-5	riken1_30e3	(Q91XC8) Similar to death-associated protein
CATGGCCGGGCGCCCCACCAG	0	15	2.41E-5	riken1_15i13	(Q99P44) Leucine aminopeptidase
CATGGGACCAACAAATAAAGC	19	4	0.0027	riken1_4o10	(P97440) Histone RNA hairpin-binding protein
CATGAAAATGTACTGTGCTAA	2	13	0.0036	riken1_20p3	(P34022) Ran-specific GTPase-activating protein
CATGTATACAGAACTGCTGGA	8	0	0.0044	riken1_2i24	(Q9UMR2) ATP-dependent RNA helicase DDX19
CATGGCCAAATTAGAGGAGTG	1	10	0.0051	riken1_32c11	(Q9YGQ1) Peptide elongation factor 1-beta
CATGCTACGCTGTGTCTGCCA	11	1	0.0062	riken1_2m14	(AAQ20009) Heterogeneous nuclear ribonucleoprotein H1-like protein
CATGCTCTCCGGTGGTACAAT	0	7	0.0070	riken1_32c11	(Q9YGQ1) Peptide elongation factor 1-beta
CATGTTGATTCCTATGCTAAA	7	0	0.0087	riken1_3a6	(Q9H165) B-cell lymphoma/leukemia 11A

# only unitags matching bursal cDNAs are listed

## BLAST results are abbreviated

**Figure 3:**
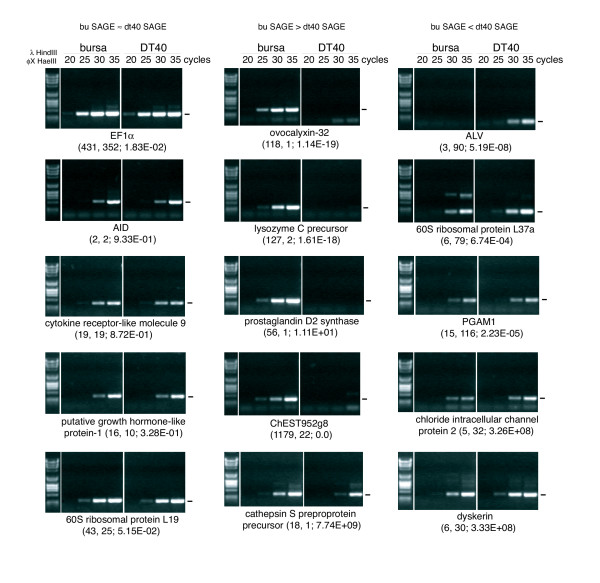
Confirmation of differential gene expression using semi-quantitative PCR. Primers derived from reference genes for SAGE tags were used for the amplification of cDNA from bursal cells and DT40 employing different cycle numbers as indicated on top of the lanes. Based on the SAGE tag counts, the reference genes were classified as likely to be equally expressed (left part), higher expressed in bursal cells (middle part) or higher expressed in DT40 (right part). The size of the expected PCR product is indicated by a bar adjacent to the gel image. The numbers of tags found for the busage and dt40sage libraries as well as the calculated significance for differential expression are indicated in brackets under the gene names.

To verify the validity of the SAGE data, semi-quantitative PCR was performed using primers close to the tags for 27 transcripts (Figure 3). This confirmed the expression pattern suggested by SAGE tag counts in the majority (21 out of 27) of the cases. Certainly, these PCR results could not be explained by the statistical variation in the SAGE data alone (FDR below 5% vs. FDR of 22% indicated by PCR). Although more analysis is needed to find out which differentially expressed genes are related to differences in the behavior of bursal B cells and DT40, the freely available SAGE repository will be a good resource to select candidates for more detailed investigations.

## Conclusions

The mapping of the SAGE tags to the recently released cDNA collections and the chicken genome has been useful to assess the completeness and accuracy of the current transcript catalog. On the positive side, it appears that the transcript build may have missed only a low percentage of genes, since relatively few tags map to genome regions far away from annotated transcription units. On the downside, fewer than 6,000 of over 19,000 tags with matches to reference sequences could be mapped to transcripts. The majority of the tags missed in transcripts are positioned downstream of annotated transcripts with a minority mapping upstream or within the genomic boundaries of transcripts. The most straightforward explanation for this is that many transcripts in the current version of the chicken transcriptome do not accurately reflect the 3' and the 5' ends of transcripts. This proposition is independently supported by the comparisons of the bursal full length cDNAs to the Ensembl transcript build which detected discrepancies to Ensembl annotated transcripts for approximately 50% of the cDNAs [10]. Another explanation for at least part of the missing transcript matches is variability in poly-adenylation and splicing, which seems to account for substantial variety in the human transcriptome [12].

Accurate definitions of the transcribed parts of the chicken genome is highly desirable not only to ascertain the correct ORFs, but also to identify transcription and translational control sequences often located in 5' and 3' untranslated regions. It should be interesting to use the genomic positions of the missed transcript tags in combination with current gene finder algorithms to improve transcript coverage. Many of the missed tags are close to already annotated exons facilitating this task. It should also be possible to use promising tag sequences to screen cDNA libraries for clones whose sequence will identify missed genes or exons. The riken1 bursal cDNA library is of excellent quality and should be suitable for this purpose.

Although the presented SAGE data provides valuable information about the expression levels of many genes in bursal cells and the DT40 cell line, the full potential of SAGE for gene expression profiling could not be exploited due to the difficulties in tag to gene assignment. Nevertheless, this first SAGE analysis in the chicken lays the basis for further studies. SAGE has the advantage that data from different experiments and laboratories are easily comparable as the tag sequences serve as a common standard. Accumulation of additional data will increasingly facilitate the interpretation of results because *bona fide *tags will be distinguished from artifacts by being replicated and even polymorphic tags will eventually be defined and assigned to their corresponding transcripts.

## Methods

### LongSAGE library construction

Total RNA from bursal tissue of chicken 20 day old CB-inbred chicks and from DT40 Cre1 cells [14] was extracted using TRIzol reagent (Invitrogen) according to the manufacturer's instructions. PolyA RNA was isolated using the mRNA DIRECT kit from Dynal . The RNA bound to oligo(dT)_25 _magnetic beads was immediately used for the construction of a LongSAGE library [1,3] following a modified protocol as described previously [15]. High fidelity PfuUltra (Stratagene) polymerase was used for the PCR amplification step. The SAGE libraries from bursal tissue and DT40 were named busage and dt40sage respectively. For each library, distinct Linker/Primer combinations were used to exclude accidental amplification of ditags from the other library.

### Sequencing of SAGE library clone inserts

The pZero-1 (Invitrogen) plasmids containing SAGE ditags as multimeric inserts were transformed into E. coli. Zeocin resistant colonies transformed by the plasmids were grown at low density on agar plates, picked and directly suspended in 50 microliters of H_2_O. This suspension was heated at 95°C for 10 minutes and stored at -20°C until further processing. The PCR amplification used primers from the plasmid backbone, M13 forward and reverse. Sequencing was performed using the Big Dye v3.1 ready reaction mix (Applied Biosystems) and a nested primer (SSP2) from the plasmid poly-linker. Reactions were analyzed on an ABI 3730 DNA Analyzer (Applied Biosystems). The raw sequencing files were processed as described previously [16].

### Ditag, tag and unitag definition

The library insert sequences were searched for ditags in which the flanking CATG tetra-nucleotides are separated by a spacer sequence of more than 31 and less than 37 bases. Ditags of identical sequence were entered only once for each library to avoid the possibility of entering PCR amplification artifacts. The ditags were then divided into two SAGE tags of 21 bases including the CATG tetra-nucleotides. The combined SAGE tag collections of both libraries were normalized to generate a collection of unitags possessing unique tag sequences. A low number of tags (197 of 129,568 total tags) were found to be identical to the sequences of the linker tags used for the library construction and therefore were removed. Care was taken to minimize the possibility of tag sequence errors by using a high fidelity polymerase for the PCR amplification step of the library construction and by rejecting any ditag sequences which contained even a single ambiguous base call or a PHRED score lower than 10. It is possible that some unitags are due to sequencing errors, but these artificial tags are unlikely to match transcript or genome sequences.

### Tag-to-gene mapping

To map the unitags to reference sequences, candidate tags were extracted from i) full length bursal cDNA sequences [10], ii) the Ensembl transcript build  and iii) the chicken chromosome sequences . Candidate tags in the transcript datasets were extracted only in the sense orientation whereas both strands of the chromosome sequences were searched. The SAGE tags, unitags and candidate tags together with relevant information concerning their positions and frequencies were entered into tables of a relational database to facilitate further analysis.

Unitag matches were sequentially searched for in the bursal cDNA collection, the Ensembl transcript build and the Genome. Once a match had been identified, that tag fell out of the remaining search process and only matches of identical sequences were accepted. To relate the position of matching unitags in the genome sequence to the Ensembl transcripts, the chromosome coordinates of the Ensembl transcripts and their orientation were extracted from their headers. The database table structure, all tabulated entries as well as the FOUNTAIN software [17] used for the analysis is freely available for download under  and .

### Calculation of the significance of SAGE count differences

To evaluate the significance of SAGE tag count differences between the libraries for each unitag, we used Fishers exact test [18] since it is most easy to use, has exact size and does not require specifying hyper-parameters like for a Bayesian approach. As usual, no method to account for multiple testing was used, so p-values were just used as a convenient tool to rank the unitags.

### Semi-quantitative PCR

cDNA was synthesized from bursal tissue and DT40 Cre1 cell line using the SuperScript Preamplification System (Invitrogen). Primers were designed to amplify a region of a few hundred base pairs encompassing the SAGE unitag sequence of the reference transcript. PCR amplification was performed using the Expand Long Template PCR System (Roche) under the following conditions: 2 min initial incubation at 93°C; 20, 25, 30 and 35 cycles consisting of 10 sec at 93°C, 30 sec at 65°C and 5 min at 68°C with 20 sec elongation per cycle.

## Authors' contributions

JMB and MBW conceived the project. MBW, RBC, HA and JMB participated in the design of the study and its coordination. MBW constructed the libraries. MBW, RBC, NH, CJ, MS, MC and YDW performed clone management, sequencing and data analysis. HA performed confirmation PCR analysis. MBW, RBC, AMK, EE, VL and JMB performed bioinformatics and statistical analysis. AMK and JMB programmed the FOUNTAIN software package to include the SAGE analysis modules. MBW, RBC, AMK, HA, VL and JMB helped draft the manuscript. All authors read and approved the final manuscript.

**Figure 1:**
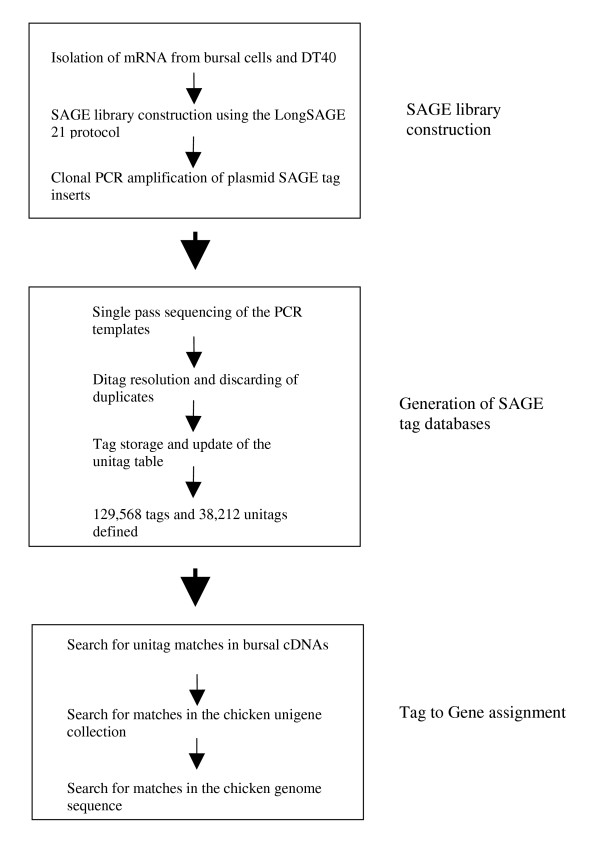
Outline of SAGE tag production and reference gene assignment.
